# PET-CT in the sub-arctic region of Norway 2010–2013. At the edge of what is possible?

**DOI:** 10.1186/s12880-015-0073-0

**Published:** 2015-08-28

**Authors:** Jan Norum, Ursula Søndergaard, Erik Traasdahl, Carsten Nieder, Geir Tollåli, Gry Andersen, Rune Sundset

**Affiliations:** Department of Clinical Medicine, Medical Imaging Research Group, Faculty of Health Sciences, UiT - The Arctic University of Norway, N-9037 Tromsø, Norway; Department of Radiology, University Hospital of North Norway, N-9038 Tromsø, Norway; Nordland hospital, N-8017 Bodø, Norway; Northern Norway Regional Health Authority trust, N-8038 Bodø, Norway

**Keywords:** PET-CT, FDG, Arctic, Northern Norway, Access

## Abstract

**Background:**

It is challenging to obtain a similar access to positron emission tomography/computed tomography (PET-CT) within the whole region served. In the subarctic and arctic region of Norway, significant distances, weather conditions and seasonable darkness have been challenging when the health care provider has aimed for a high quality PET-CT service with similar availability to all inhabitants.

**Methods:**

The PET-CT service at the University Hospital of North Norway (UNN) was established in May 2010. The glucose analogue tracer fluorine-18 fluorodeoxyglucose (FDG) was delivered from Helsinki, Finland. An ambulatory PET-CT scanner was initially employed and a permanent local one was introduced in October 2011. In March 2014, we analysed retrospectively all data on the PET-CT exams performed at the Section of Nuclear Medicine, Department of Radiology during a 32 months time period 2010–13. The following patient data were recorded: gender, age, diagnosis, residence and distance of travelling. There were in total 796 exams in 706 patients.

**Results:**

Four hundred sixty-one PET-CT exams per million inhabitants were, on average, performed per year. Lung cancer (32.7 %), malignant melanoma (11.3 %), colorectal cancer (10.9 %) and lymphoma (9.7 %) constituted two-thirds of all exams. Three-fourths were males and the median age was 63.5 years (range 15.2–91.4 years). The access to PET-CT exam varied within the region. The southern county (Nordland) experienced a significantly less access (*p* < 0.0001) to the regional service. Except for malignant melanoma, this finding was observed in all major cancer subgroups. In colorectal cancer and lymphoma a lower consumption of PET-CT was also observed in the northeastern county (Finnmark). Patients’ mean distance of travelling by car (one way) was 373 km (median 313 km, range 5–936 km).

**Conclusion:**

PET-CT was not similarly available within the region. Especially, inhabitants in the southern county experienced less access to the regional service. National and regional standards of care, new scanners and improved collaboration between hospital trusts may alter this situation.

## Background

The integral role of PET using FDG in the staging of several malignancies and in the evaluation of inflammatory disease has been established [1]. There has been an increasing interest in the prognostic and predictive role of FDG-PET scans. Studies have shown that absence of metabolic response to neoadjuvant therapy correlates with poor pathologic response.

The need for PET/CT scanner installations in Norway was first launched in 2000 [2]. Health care decision makers have since then ordered in total six Health Technology Assessments (HTAs). The early funding was made possible in 2004 through collaboration between the Ministry of Education and Research, Norwegian Research Council and Amersham Health AS [3]. Somewhat surprising, the Ministry of Health and Care Services was not present among the financiers. The first PET-CT was located in Oslo (southeastern region) and the financiers stated that the new diagnostic tool should be of benefit to all Norwegian health regions. In 2009, the Norwegian Knowledge Centre calculated the necessary number of PET-CT scanners to serve the Norwegian population [4]. They concluded the figure to be between 4 and 14 PET-scanners, depending on whether this technology was implemented in radiation planning or not [4]. At this time indications for PET-CT were lung cancer and Hodgkin’s lymphoma.

Following the introduction of the PET-CT modality in Norway, it became clear from the early evaluations that the availability to this service was dropping with the distance to the PET-centres. This was especially observed in northern Norway. To counteract this tendency and improve the service to the population of Northern Norway, the University Hospital of North Norway (UNN) initiated a process to implement a PET-CT scanner in Tromsø. Northern Norway covers almost half of Norway’s land mass and is about two-thirds of the size of the UK, but the population is only 470,000 inhabitants. They live in three counties named Finnmark, Troms and Nordland and 2500 people live in the Norwegian arctic, mainly on the Svalbard islands. Significant distances to national health care facilities in southern and western Norway and health care facilities within northern Norway have been a constant challenge to the Northern Norway Regional Health Authority (NNRHA) trust. The northern area has a subarctic and arctic climate. Cold and rough weather conditions, long distances, seasonable darkness and snow have to be handled. Consequently, it is of importance to clarify whether a PET-centre in this remote geographical area, outside major cities and Ivy League academic institutions, can offer equal availability of PET services regardless of geography and socio-economics.

## Methods

### The service analysed

**Fig. 1:**
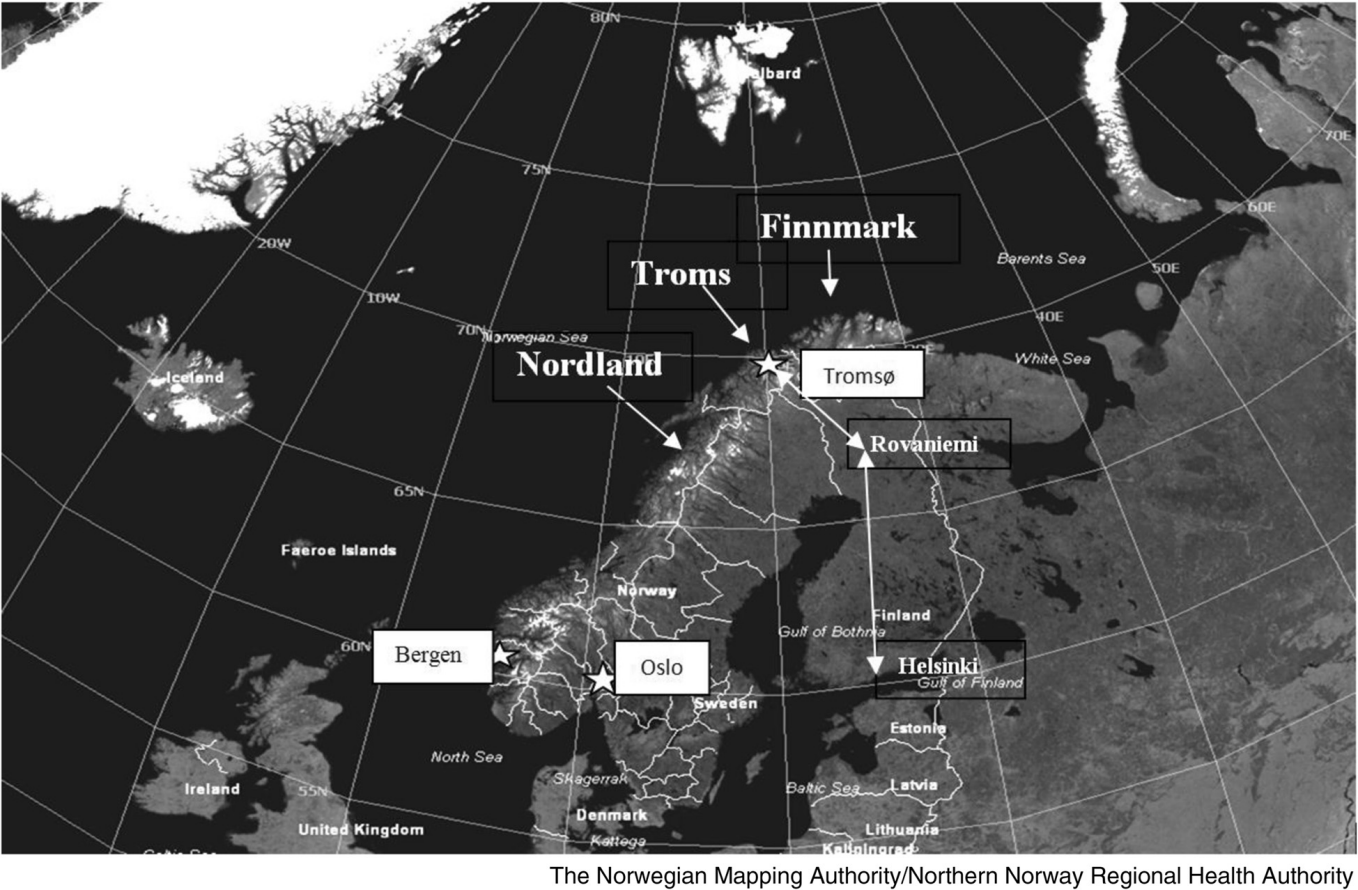
The Scandinavian peninsula with Norway. The route from Helsinki to Tromsø is shown. White stars marks cities in Norway with PET-CT scanners (Bergen, Oslo, Tromsø). The white lines illustrate the borders between counties in Norway and Sweden. The map was provided and given permission to reproduce by The Norwegian Mapping Authority/Northern Norway Regional Health Authority

The evolution of nuclear medicine during the early days of this millennium pointed clearly to the need for a PET–CT scanner in the northern region. However, the University Hospital of North Norway’s location at the top of Europe and its limited economic resources called for a cost-effective service. The city of Tromsø is situated at the centre of northern Norway with more than 800 kilometres (km) by car to the southern border of Nordland County and 900 km to the Russian border in the east of Finnmark County. Details are shown on the map in Fig. 1. The distances from Tromsø to the nearest national cyclotrons (Bergen 1809 km and Oslo 1641 km) are significant. Consequently, the best logistics were obtained through cooperation with the Finnish company MAP Medical Technologies OY in Helsinki. The distance is 1355 km. FDG was produced in Helsinki in the early morning 05.40 a.m. Finnish time (04.40 a.m. Norwegian time) and transported by Finnair® to the city of Rovaniemi in northern Finland. The tracer was then transported further by airplane to Tromsø by Oulun Tilauslento OY. The batch reached Tromsø 5 h later at 09.40 a.m. local time and could be used until 3 p.m. To make this transportation possible (2.75 half-lives of F-18 FDG), the activity when ready for transportation was 45 GBq. The activity when arriving our lab in Tromsø was 6.7 GBq. The first patient was examined in May 2010 and the capacity was 8 patients every Wednesday. Initially, Alliance Medical served several hospitals in Scandinavia employing a PET-CT placed in a semitrailer. This trailer was driven according to a specific route visiting hospital in Tartu and Tallin in Estland, Kuopio and Oulu in Finland, Tromsø in Norway and Umeå, Falun and Örebro in Sweden. The semitrailer solution was replaced in October 2011 by a stationary PET-CT scanner. In November 2012 a permanent PET-CT service was established. This was made possible by a private donation by businessman Trond Mohn.

### Data included

Data included all consecutive PET-CT exams performed at the Section of Nuclear Medicine Department of Radiology UNN. There were in total 796 exams in 706 patients. In April 2014 all data were retrospectively analysed. The 4 years time period 2010–13 was selected and the following data were recorded: gender, age, diagnosis, number of exams, place of living and distance of travelling. Inhabitants of Svalbard (three patients) were included in the Finnmark figures. However, patients living on the Svalbard islands were not included when the distance to Tromsø was calculated. Distance was calculated in kilometres (km) from the community of each patient to the UNN in Tromsø employing the NAF converter (www.naf.no). The converter measured the distance by road. In case of alternatives, the shortest distance was chosen.

The mean number of inhabitants 2010–2013 in the three counties (Finnmark, Troms and Nordland) of northern Norway was calculated according to Statistics Norway (www.ssb.no). The mean figures were 73,649, 158,279 and 237,871 inhabitants, respectively.

### Quality control, statistical analysis and authorisation

Individual data were recorded from the database at the Section of Nuclear Medicine, Department of Radiology, UNN. Microsoft Excel Mac 2011 was used for the final database and the IBM SPSS Statistics 2011 was used for calculations and statistical analysis. Descriptive statistics and Chi-squared test for 2×2 tables were used for the comparison between subgroups. Significance was set to 5 %. The study was carried out as a quality of care analysis and consequently no ethical committee or Data Inspectorate approval was necessary. Similarly, no approval from the Regional Committees for Medical and Health Research Ethics (REK) or from the Norwegian Social Science Data Services (NSD) was necessary.

## Results

During the early years (2010–2011) a total of 7.5 out of 30 planned days of PET-CT scanning were cancelled (25 %) between 9th of August and 15th of April. The reason was technical scanner failure. The ambulatory PET-CT scanner transportation on rough Nordic winter roads was clearly a risky business. Following the establishment of the permanent local PET-CT scanner, these early problems were solved.

During study period a total of 796 exams were performed in 706 patients (1.13 exams/patient). The highest re-examination rate (1.5) was observed among malignant melanoma patients. The figures constituted annually 461 PET-CT exams per million inhabitants and lung cancer (32.7 %), malignant melanoma (11.3 %), colorectal cancer (10.9 %) and lymphoma (9.7 %) constituted two thirds of all exams. Details are shown in Table 1.

**Table 1:** An overview of PET-CT scans performed and number of patients examined (2010–2013)

		Exams		Patients		
Variable		PET-CT	%	PET-CT	%	Exams/pt
Total		796	100	706	100.0	
Sex	Females	324	40.7	291	41.2	1.1
	Males	472	59.3	415	58.8	1.1
Year	2010	126	15.8			
	2011	202	25.4			
	2012	203	25.5			
	2013	265	33.3			
County	Finnmark	156	19.6	140	19.8	1.1
	Troms	349	43.8	311	44.1	1.1
	Nordland	282	35.4	249	35.3	1.1
	Others/unknown	9	1.1	6	0.8	1.5
Diseases	Lung cancer	260	32.7	246	34.8	1.1
	Malignant melanoma	90	11.3	61	8.6	1.5
	Colorectal cancer	87	10.9	76	10.8	1.1
	Lymphoma	77	9.7	71	10.1	1.1
	Head & neck carcinoma	44	5.5	40	5.7	1.1
	Urogenital cancer	28	3.5	21	3.0	1.3
	Thyreoid carcinoma	25	3.1	23	3.3	1.1
	Gynaecological cancer	25	3.1	21	3.0	1.2
	Upper GI-cancer	17	2.1	17	2.1	1.0
	Breast cancer	8	1.0	8	1.1	1.0
	Other cancers	14	1.6	13	1.8	1.1
	Cancer, unknown origin	54	6.8	46	6.5	1.2
	Inflammatory disease	51	6.4	44	6.2	1.2
	Sarcoidosis	12	1.5	11	1.8	1.1
	Others	4	0.5	2	0.3	2.0

There was no significant difference in age between genders. The mean age among females and men were 59.8 years (median 62.6 years, range 20.8–85.8 years) and 61.8 years (median 64.9 years, range 15.2–91.4 years), respectively.

Whereas the counties of Finnmark and Troms generally had a similar access to PET-CT, the population of Nordland County experienced a different situation. During study period, the ratio (percentage of PET-CT scans/population) was 0.7 and 1.3 (Table 2) (*p* < 0.0001), and the number of patients with PET/CT exams during the 4 year period differed significantly between the three counties (*p* < 0.0001). The proportion of the population undergoing a PET-CT scan in each northern county during study period were Nordland 0.10 %, Troms 0.20 % and Finnmark 0.19 %, respectively. Looking at the four major cancer groups, a similar availability in all counties was only observed in malignant melanoma. The greatest difference was seen in the lymphoma group. Details are shown in Table 2. Due to low numbers of cancer patients with unknown origin, we did not run any comparison between counties in this setting.

**Table 2:** The ratio of PET-CT exams/population in total and in each county of northern Norway is shown. The figure of all three northern counties was set as 1.00. (For example: All exams 787 exams/470,000 inhabitants = 0.17 % = 1.00)

	Nordland	Troms	Finnmark
Lung cancer	0.57	1.34	1.66
Malignant melanoma	0.99	1.02	0.99
Colorectal cancer	0.88	1.25	0.84
Lymphoma	0.67	1.62	0.74
All exams	0.71	1.32	1.26

The mean distance (one-way) from patients’ place of living to Tromsø (by car) was 373 km (median 313 km, range 5–936 km). The corresponding figures in each county were: Finnmark 610 km (median 568 km, range 397–863 km), Troms 130 km (median 123 km, range 5–332 km) and Nordland 545 km (median 550 km, range 244–936 km).

## Discussion

In this study, we have documented that the number of PET exams in northern Norway increased from 268 in 2010 to 564 exams/million population in 2013. These figures are low compared to Norway in general and neighbouring countries. The finding must be seen in the context of relatively few PET-CT scanners in Norway and especially in our region. The number of PET-CT scanners per million inhabitants in Europe varied in 2009 between 0.3 (Ireland) and 3.9 (Denmark) and the Norwegian figure was 0.8 [4]. In the Nordic countries, the first PET facilities were established in Sweden in the late 1970s followed by Finland in 1988 and Denmark in 1989 [5]. Since the introduction of PET at the Norwegian Radium Hospital in Oslo back in 2005, the total number of PET-exams in Norway has increased from 80 in 2005 to 7525 in 2014. Employing the national population data of 2013 (www.ssb.no) (population 5.1 million), the corresponding national annual number of exams per million was calculated as 1475. Following the implementation of a PET scanner in Bergen (western Norway) in 2009, the figures of the western region rose from 293 exams in 2009 to 1616 exams in 2014 (personal communication dr. Michael Biermann, Haukeland University Hospital, Bergen Norway). With a population of 1074 million (www.helse-vest.no), the 2014 figure of the western region can be calculated as 1504. Similar findings may be indicated employing the data of Høilund-Carlsen and colleagues [5] who reported a total of 6056 PET/CT examinations performed between 2006 and 2009 (3.8 years) in the Region of Southern Denmark (1.2 million inhabitants). According to data from Stevens presented at the annual meeting of the European Association of Nuclear Medicine (EANM) in 2011, PET examinations per million population in 2010 ranged within the Western-European countries from around 1000 in U.K. and Eire to 4000 in Italy (www.auntminnieeurope.com).

Looking to Canada, the Canadian Institute of Health Information (www.cihi.ca) reported 62,668 PET-CT exams performed in Canada in 2011–2012 giving a number of exams/million of 1800 (34.8 million inhabitants). Around 20 % of the worldwide PET/CT installed base is located in Europe [6]. Bedford and Maisey [7] calculated an increasing need for PET scanners. In their mathematical model, (employing U.K. data) the need was 2026 PET exams/million population to cover all indications in cancer care. Based on all these data, an underuse of PET-CT in our region can be strongly indicated.

Furthermore, the regional service in northern Norway was not equally accessible to all inhabitants within the region. Inhabitants of Nordland County experienced a significantly lower access rate. During the time period 2010–2012, a possible learning curve was indicated for the clinicians in referring or not referring to PET. However, data for 2013 were again deviating. A follow up study including 2014–15 should be done to clarify whether any learning curve can be concluded.

The distance from patients’ place of living to the PET centre in Tromsø was significant. This is due to the fact that the limited population (0.47 million) is scattered within a significant geographic area (174,000 km^2^).

The most common diagnoses among patients undergoing PET-CT scan in our region were lung cancer, malignant melanoma, colorectal cancer and lymphoma. PET-CT has been documented the best non-invasive technique for evaluation of lymph nodes and extrathoracal disease in lung cancer [8, 9]. Despite the fact that PET in the management of non-small cell lung cancer (NSCLC) has been clearly documented and included in the national guideline [10], significant variations in the pattern of use within northern Norway was disclosed. The Norwegian guideline state that PET-CT is recommended in all NSCLC patients considered candidates for curative treatment. When small peripheral lesions (stage 1) are revealed, patients may be referred to the multidisciplinary team (MDT) and undergo surgery without any preoperative examination. The place of preoperative PET-CT in small cell lung cancer (SCLC) has been less documented [11], but suggested in the national guidelines. Langer and colleagues [12] did not observe any place for PET-CT in response evaluation in lung cancer.

Differences between counties in our region could be due to referrals from Nordland to the southeastern or western PET centers in Norway. There could also be a “leakage” of patients from the UNN due to waiting list/time and episodes when the local scanner was out of service. We therefore contacted the hospital trusts and got information from their Department of Economics about all PET-CT scans paid for during study period. This revealed that the UNN had paid for 89 PET-CT exams (2010–13) outside northern Norway. Similarly, Nordland hospital had paid for 41 exams (2010–12). Correcting for these figures, the significant less use of PET-CT among patients in Nordland was still present.

Another fact may be differences in waiting time. Whereas the MDT at the UNN had weekly appointments reserved at the PET-CT scanner, this was not the fact for the MDT at Nordland hospital. In the national guideline it is recommended that PET-CT exams should not delay curative surgery by more than 2 weeks. Employing our data, we could not detect whether patients in Nordland underwent more frequently curative surgery without any preoperative PET-CT due to long waiting lists. However, due to our findings, we have now reserved weekly appointments in Tromsø for the Nordland hospital’s MDT.

The benefit of PET-CT in the follow up of malignant melanoma has been debated. Recently, Danielsen and co-workers [13] performed a systematic literature search and identified 7 original studies on the diagnostic value of FDG-PET in the follow-up of cutaneous malignant melanoma (CMM). The mean sensitivity of PET was 96 % and the specificity was 92 %. The positive and negative predictive values were, respectively, 92 and 95 %. They concluded PET had a high diagnostic value and this indicated utility in the routine follow-up program. However, the number of prospective studies of high quality was scarce. The Norwegian guideline for the diagnosis and follow up of malignant melanoma presupposes that the patients are well evaluated (including ultrasound, CT, MR or PET-CT) prior to surgery. Especially prior to operation on regional lymph node metastases or prior to resection of distant metastasis, PET-CT has been considered useful [14–16]. The three northern counties (Nordland, Troms and Finnmark) had similar age adjusted incidence rates of malignant melanoma (2007–2011) per 100.000 person-years [17]. Details are shown in Table 3. Looking at these figures, it was satisfactory to observe the equal access to PET-CT among this group of cancer patients. The strong cooperation within plastic surgery between the two major hospitals located in Bodø and Tromsø and the ambulatory service from Tromsø to Bodø (by plastic surgeons) may explain this similar standard of care. We searched for data on PET and malignant melanoma from other Norwegian regions, but revealed only one unreported study from 2007 (Oslo University Hospital) concluding the disease accounted for 3 % of all PET exams. Due to different incidence rates and later updates of the national guideline [16], this data is not comparable to our findings.

**Table 3:** The table shows the age adjusted incidence and mortality rates per 100,000 and 5-years relative survival of all cancer, lung cancer, malignant melanoma, colon cancer and non-Hodgkin lymphoma (NHL) in Norway and each county in northern Norway. The figures are based on data from the Norwegian Cancer Registry for 2009–2013 [17]

Cancer	Region	Incidence		Mortality		5-years surv.	
		F	M	F	M	F	M
All	Norway	302.0	374.1	85.9	115.5	68.9	68.5
	Nordland	284.1	351.9	84.0	116.0	67.2	68.5
	Troms	276.9	353.6	83.6	106.2	66.7	70.7
	Finnmark	258.9	333.4	80.2	129.7	66.9	58.2
Lung	Norway	26.0	34.9	17.6	26.2	18.8	13.1
	Nordland	23.4	33.5	18.5	25.3	17.0	11.8
	Troms	24.2	33.9	18.2	24.2	17.0	17.1
	Finnmark	26.4	51.2	19.4	38.3	20.6	11.9
Mal.mel.	Norway	21.1	20.2	2.6	4.3	88.3	79.0
	Nordland	11.6	12.5	2.1	3.0	75.7	77.8
	Troms	14.3	12.5	2.0	2.9	91.9	87.3
	Finnmark	11.2	8.9	1.7	1.9	88.7	74.0
Colon	Norway	24.6	27.1	9.0	11.0	63.3	59.5
	Nordland	24.8	27.7	8.2	10.6	64.9	63.5
	Troms	22.8	25.4	8.5	9.9	64.9	66.3
	Finnmark	21.7	19.3	5.4	8.6	77.3	48.5
NHL	Norway	9.6	13.2	1.9	3.4	76.4	70.5
	Nordland	11.0	12.7	2.7	3.3	82.4	75.8
	Troms	9.5	11.5	1.9	2.5	88.5	77.2
	Finnmark	9.3	13.5	1.5	2.6	76.2	66.8

*F* female, *M* male

According to the national guideline [18], PET-CT has so far no place in primary evaluation of patients diagnosed with colorectal carcinoma (CRC). At present, PET-CT has been reserved for the examination of patients with suspected local recurrence when other diagnostic tools have been inconclusive. It is also recommended in the preoperative setting when curative resection of liver metastases has been planned. This is in coherence with our finding that CRC constituted only 10.8 % of the patients examined despite this disease is the most common cancer in Norway. However, different use of PET exams between counties should be further explored. It could be speculated that differences may be due to later presentation to the health care system due to less general accessibility and less availability to early diagnostics like endoscopy. However, the 5 years relative survival of colon cancer does not support such a statement (Table 3). The northern region has similar results as other health regions, according to national quality data (www.helsenorge.no) [17]. In the future, there are reasons to believe PET-CT may be more commonly used in CRC due to advocates for the implementation of this diagnostic tool in the follow up program. Recently, a cohort of 132 patients, treated by surgery with curative intent, was included in a follow-up analysis [19]. Patients were followed prospectively with scheduled controls at 3, 6, 12 and 24 months after curative surgery. The controls included CEA, chest X-ray, ultrasound, CT and PET supplemented by clinical examination. The end-point was recurrence. Sensitivity and specificity was estimated 2 years after surgery. Twenty-five patients experienced recurrence, detected at scheduled controls (*n* = 18) and at intervals between them (*n* = 7). The results of CT and PET were correlated with recurrence. CT combined with PET had the highest specificity and sensitivity. A total of 72 % of recurrences were detected at scheduled controls. The findings supported a strict follow-up program following curative surgery for colorectal cancer. The authors suggested that FDG-PET combined with CT should be included in control programs.

The fourth major patient group in our study was lymphoma [20]. According to the national guideline, PET-CT was requested in research settings, for example in response evaluation [20]. The focus on PET-CT in the research setting may reflect the significant “overuse” of PET-CT in the county (Troms) being the host of the University hospital. Several researchers have published the potential prognostic impact of PET-CT in lymphoma [21–23]. Gallicchio and colleagues [21] evaluated the prognostic significance of standardized uptake value (SUVmax), metabolic tumor volume (MTV), and total lesion glycolysis (TLG) obtained by FDG PET-CT in patients with diffuse large B-cell lymphomas (DLBCL). The SUV may be calculated either pixel-wise yielding a parametric image, or over a region of interest. This may be done for any image acquired at time point *t*, or for all images of a dynamic series acquired at multiple time points. MTV may be derived from SUV images and may be of interest for both target volume definition in radiotherapy and monitoring response to therapy. TLG may be defined as (SUV_avg_) × (tumor volume), with a threshold of 45 % SUV_max_ in the volume of interest. Kaplan-Meier survival analysis for SUVmax showed a significantly better event free survival in patients presenting higher values as compared to those with lower values. In Hodgkin lymphoma patients, routine PET-CT in first remission has not been shown superior to clinical follow-up for patients with no residual mass [24].

PET-CT was also used in carcinoma of unknown primary (CUP) in our study. We did not compare the three northern counties with regard to CUP due to low numbers (54 patients). Breuer and colleagues [25] have published the benefit of PET-CT in this setting. They concluded FDG PET-CT a helpful tool for the identification of the primary tumor in patients with CUP. In 26 % of the patients, a primary tumor was identified. The tool was also able to provide an accurate assessment of prognosis based on the extent of the disease without the need for identification of the primary tumor. Kaplan-Meier analysis revealed 3-year survival rates of 73 % (without evidence of malignancy), 71 % (locoregional disease), and 23 % (extensive disease), respectively.

Our study was not intended as a clinical paper on diagnostic performance. We focused on basic demographics of northern Norway. We did not include/assess clinical data on incidence numbers and disease stages at diagnosis in the three regions compared to the rest of Norway. However, incidence data are available from the Cancer Registry of Norway [17]. As illustrated in Table 3, there are some differences. Lung cancer is more common in Finnmark and malignant melanoma is less common in northern Norway. However, these differences cannot explain the variations in accessibility to PET-CT within our region.

Hybrid PET-MRI scanners are now available for clinical use. PET-MRI combines the unique features of MR imaging including excellent soft tissue contrast, diffusion-weighted imaging, dynamic contrast-enhanced imaging and other specialized sequences as well as MRI spectroscopy with the quantitative physiologic information that is provided by PET. There are potential competitive advantages (for example the radiation dose) of PET-MRI over PET-CT. In the future, we therefore plan for a PET-CT and a PET-MRI service and a cyclotron located in Tromsø and running from 2017. Furthermore, a PET-CT scanner has been planned at Nordland hospital in Bodø. Hopefully these plans together with a follow up of this study may improve the equal availability of PET services in our region.

## Conclusion

PET-CT was not similarly accessible within the region. Especially, inhabitants in the southern region experienced less access to the service. National and regional standards of care and improved collaboration between hospital trusts may alter this situation. It was possible, but challenging to run a PET-CT service in the northern sub-arctic region of Norway. An ambulatory scanner is not recommended. In the future a local cyclotron is preferable.

## Abbreviations

UNN: University Hospital of North Norway
NNRHA: Northern Norway Regional Health Authority
